# Need for Affect, Problematic Social Media Use and the Mediating Role of Fear of Missing Out in European and Arab Samples

**DOI:** 10.2147/PRBM.S435437

**Published:** 2023-12-14

**Authors:** Areej Babiker, Mohamed Basel Almourad, Constantina Panourgia, Sameha Alshakhsi, Christian Montag, Raian Ali

**Affiliations:** 1 College of Science and Engineering, Hamad Bin Khalifa University, Doha, Qatar; 2 College of Technological Innovation, Zayed University, Dubai, United Arab Emirates; 3 Department of Psychology, Bournemouth University, Poole, UK; 4 Department of Molecular Psychology, Institute of Psychology and Education, Ulm University, Ulm, Germany

**Keywords:** problematic social media use, need for affect, affect approach, affect avoidance, fear of missing out, cross cultural

## Abstract

**Purpose:**

The growing awareness and concern about the excessive use of social media have led to an increasing number of studies investigating the underlying factors contributing to this behavior. In the literature, it is discussed that problematic social media use (PSMU) can impact individuals’ mental health and well-being. Drawing on the Interaction of Person-Affect-Cognition-Execution (I-PACE) model, this study aimed to examine the association between the need for affect (affect approach and affect avoidance) and PSMU (operationalized via the social media disorder scale), as well as the mediating role of fear of missing out (FoMO) in that relation.

**Participants and Methods:**

Data were collected via an online survey from 513 participants in European and Arabic countries. Regression and mediation analyses were conducted to explore the relationships between affect approach, affect avoidance, FoMO, and PSMU.

**Results:**

Regression analysis results indicated that both affect approach and affect avoidance as part of the need for affect construct significantly predicted PSMU in both cultural contexts. Mediation analysis showed that FoMO partially mediated the relationship between affect approach and PSMU in the Arab sample but not in the European sample. Beyond this, FoMO partially mediated the relationship between affect avoidance and PSMU in both cultural samples.

**Conclusion:**

The present study indicates that managing emotions could be an effective strategy to combat PSMU. In line with this and against the background of the data business model behind social media companies, we deem it to be of importance to minimize triggers related to FoMO in the design of social media platforms (for example, push notifications). This might be particularly relevant for individuals with a high inclination towards affect approach and affect avoidance.

## Introduction

Social media has emerged as a powerful communication medium allowing to build social capital^1^ and it has become an integral and ubiquitous part of people’s lives with nearly five billion users worldwide.^2^ Despite its positive effects on the lives of individuals, concerns have arisen regarding the compulsive or problematic use of social media, commonly referred to as “social media addiction”,^3^,^4^ but the terminology has been criticized.^5^ Concerns around excessive social media use primarily stem from potential adverse consequences. It is discussed that excessive use of social media might impact on mental health,^6^,^7^ productivity at work^8^ and to some extent, subjective well-being.^9^ Individuals may exhibit various signs akin to addictive behaviors, including mood modification, salience, tolerance, withdrawal symptoms, conflict, and relapse, in their relationship to social media use.^7^

Previous research has posited that engagement with social network sites (SNS), like Facebook, elicits positive affective experiences characterized by heightened positive valence and arousal levels.^10^ Conversely, intensive social media use has frequently been viewed as a coping mechanism employed to address adverse emotional states and encounters^11^ (see also the more general work of Kardefelt-Winther^12^ on this topic). For instance, individuals resort to social media platforms to alleviate negative mood,^13^ to combat feelings of boredom and loneliness^14^ and to get distracted from offline difficulties or responsibilities linked to negative emotions.^15^,^16^ Furthermore, research shows that individuals lacking social support tend to escape in the virtual realm of social media.^17^ These findings suggest that people may turn to social media both to experience new affective states in the online realm and to escape from challenging emotional experiences in the offline world. In other words, this highlights the potential for leveraging social media data to gain insights into an individual’s need for affect.^18^

The concept of the need for affect (NFA) is considered as a stable intrinsic trait^19^ and pertains to individual differences in the motivation to approach or avoid emotion-inducing situations.^20^ NFA is distinguished from the concept of emotion regulation and refers to people’s orientations or attitudes towards seeking or avoiding experiencing emotions.^19^ It has been extensively studied to understand individual differences across multiple domains such as reactions to media and entertainment,^21^ consumer’s behaviors^22^ and social perception.^23^ Additionally, NFA has been found to impact the selection and processing of information, thereby influencing attitude development and behavioral regulation.^24–26^ Consequently, NFA could play a pivotal role not only in understanding individuals’ engagement with social media but also in exploring problematic use of social media itself.

Individuals with varying levels of NFA exhibit distinct patterns of emotional expression in social media. Notably, users with higher NFA seem to be more inclined to express their emotions on online platforms^18^, and actively search for more emotional websites.^27^ However, the link between NFA and PSMU remains understudied in the existing literature. Previous research has primarily focused on the role of personality traits, particularly neuroticism,^28^ which is closely related to NFA but differs in its emphasis. This is because NFA measures the extent to which individuals are inclined to seek out (affect approach) and to avoid new emotional experiences (affect avoidance), but not their susceptibility to experiencing negative emotions. On the other hand, neuroticism refers to an individual’s emotional instability and tendency to experience depression and negative affect.^29^ As indicated by Marino et al,^30^ two key motives for addictive social media usage are to enhance positive affect or, to cope or diminish negative effect. Hence, NFA may hold predictive potential and contribute to the understanding of PSMU development.

Another concept that is closely linked to the understanding of PSMU development is the fear of missing out (FoMO). FoMO is characterized by a prevailing sense of worry or anxiety that others may be experiencing enjoyable or rewarding experiences without one’s presence.^14^,^31^ It is noteworthy that it is debated if FoMO has a more trait or state like character.^32^,^33^ Aside from this discussion, it has been put forward that FoMO can be stimulated and amplified by social media design and usage,^34^ hence the industry behind social media products uses design elements to prolong online behavior^35^,^36^ also via the psychological FoMO process. For instance, the constant influx of notifications on social media platforms can create a sense of urgency in individuals, compelling them to frequently check their devices in an attempt to alleviate the fear of missing out. Although FoMO strongly correlated with self-reported problematic smartphone use (PSU) severity, it did not show associations with objectively measured smartphone use.^37^ Paradoxically, even muting social media notifications can exacerbate the feeling of FoMO, as individuals might perceive that they are missing out on important updates.^38^

Consequently, heightened feelings of FoMO can result in problematic social media use, and can even be associated with depression, anxiety, and neuroticism.^39^ The study conducted by Fioravanti^39^ suggested that some individuals may be driven toward excessive use of social media due to the need to get in touch and maintain control of their online presence and interactions. In the light of potential negative effects of FoMO on well-being, research by Alutaybi et al^40^ has proposed various strategies to mitigate its impact. These approaches go beyond simple notification settings and include measures such as setting appropriate social expectations, providing summaries and wrap-ups of social events, using specialized notifications to indicate content urgency, and recording temporarily available content for later viewing. Another line of research on FoMO has unveiled significant associations between FoMO and negative affectivity.^41^,^42^ Additionally, it has been demonstrated that FoMO exhibits an inverse correlation with life satisfaction.^43^ Given that NFA is conceptualized as individual’s orientation towards emotional experiences, a possible assumption emerges that FoMO can be a natural consequence of this tendency, consequently leading to PSMU.

In this study we adopted the I-PACE process model, which proposes the role of the Interaction between Person-Affect-Cognition-Execution in problematic internet use.^44^ In this context, the individual trait NFA, relates to how individuals engage with content and interactions on social media platforms, driven by their preferences for emotional experiences. FoMO on the other hand, comprising affective and cognitive facets, may increase the motivation to stay connected, engage frequently, and react to notifications to avoid missing important moments on these social media platforms. FoMO can potentially act as a mediator, connecting deficits in psychological needs with social media engagement.^14^ The interaction between these factors, ie, personality, affect, cognitive processes and behavioral execution (social media use) influences the likelihood of engaging in problematic use behaviors.

In addition, researchers examining the constructs of FoMO, addictive social media use and affect expression on social media have observed differences related to age and gender in their findings. For example, a study^45^ revealed that males exhibited significantly higher levels of both FoMO and addictive social media use compared to females. However, another large-scale-study^33^ reported no gender-related differences in the experience of FoMO. Additionally, other researchers^46^,^47^ revealed that females display higher levels of PSMU, a condition being linked to higher FoMO (eg, see associations with problematic WeChat use).^48^ Regarding emotional expression on social media, a study^49^ investigated emotional expressions within comments on the MySpace platform; their findings revealed a propensity for females to provide and receive more positive comments compared to males. This observation may imply that females may show a greater efficacy as social network site users due to their ability to leverage positive affect.

Regarding age-related differences, a study by Rozgonjuk et al^33^ reported that younger participants had higher FoMO scores, while study by Barry and Wong^50^ found no age cohort differences in overall FoMO. Furthermore, Andreassen et al^51^ found an inverse relationship between age and addictive social media use, hence again younger age was associated with higher PSMU, something also backed up by a recent meta-analysis.^52^ In this context, a large-scale cross-sectional study^51^ also revealed that female participants reported higher levels of addiction to social media usage compared to males. The existing literature suggests the necessity for further exploration, especially concerning the potential effect of age and gender on individuals’ experiences with FoMO and PSMU, as well as their association with the need for affect.

In general, there has been increasing research examining the deleterious effects of PSMU on wellbeing.^9^ In response to these concerns, researchers have endeavored to gain a comprehensive understanding of the underlying motivations driving users’ engagement with social media platforms. It is evident that affective factors play an important role in shaping this behavior; individuals often turn to social media platforms as a means to encounter new affective states or to escape from negative affective experiences.^18^ We hypothesize that those with higher NFA are more likely to exhibit FoMO which in turns could lead to PSMU as well. Moreover, the findings of a recent study,^53^ suggest that emotion dysregulation and FoMO play important roles as affective and cognitive mechanisms associated with problematic social media use (PSU), with FoMO mediating the relationship between impulse control and the severity of PSU. Additionally, when people worry or fear missing out on what’s happening on social media, they may end up spending more time there, which could increase the likelihood of PSMU. This worry or fear might not only be caused by how social media is designed but could also be related to an individual’s NFA trait. Hence, given the robust correlation between FoMO and PSMU^39^ and the fact that FoMO involves attempts to regulate and mitigate negative emotional states,^31^ we hypothesize that FoMO would mediate the relationship between NFA and PSMU.

The experience of FoMO could play a significant role in interpreting the relationship between NFA and PSMU. Therefore, by controlling for FoMO, we may be able to reduce or even eliminate the impact of NFA on the development of PSMU.

Hence, this study aims to address the following research questions:

1. Is there an association between age, gender, affect approach, affect avoidance, FoMO and PSMU?
2. Can the need for affect components, affect approach and affect avoidance, predict an individual’s PSMU?
3. Does FoMO mediate the relationship between affect approach, affect avoidance, on one hand and PSMU, on the other?

Furthermore, by recruiting samples from two distinct cultural backgrounds, European and Arab, the present study aims to explore whether the association between NFA and PSMU, mediated by FoMO, holds true across different cultures. This is of relevance, because psychological research is hampered by the WEIRD problem.^54^ WEIRD describes that much research in psychology has been carried out in Western, Educated, Industrialized, Rich and Democratic samples.^55^ Therefore, it is of high importance to see if findings are robust across diverse samples. Demonstrating the impact of NFA on PSMU will contribute to the literature on the role of personal differences and psychological factors in the prevalence of PSMU. This research will aid in designing interventions that consider the affective aspect of personality in its two counterparts, avoid and approach. By highlighting the mediating role of FoMO, interventions can focus on reducing FoMO triggers and weakening the impact of NFA on PSMU. For instance, implementing measures suggested by Alutaybi et al^40^ would be more effective for individuals with high NFA, allowing for personalized interfaces and specialized interventions. These mitigations have the potential to address the specific needs of individuals and promote healthier social media usage.

## Materials and Methods

### Participants and Procedures

Participants were recruited from European and Arab countries utilizing the Prolific (www.prolific.com) and Cint (www.cint.com) online platforms, specialized in obtaining respondents for research studies, including surveys. The European countries included were from Germany, Denmark, Finland, the Netherlands, Norway, Sweden, and Switzerland. The Arab countries were all considered in the sampling, as will be presented later. This selection of countries in each of the two groups was based not only on their classification as European vs Arab but also on their cultural similarity, as determined by the World Values Survey (WVS),^56^ a comprehensive cross-national study examining attitudes and values across various cultures. The WVS created a cultural map that groups similar countries based on two dimensions: secular-rational values and self-expression values that were selected out of ten indicators using factor analysis.

Before distributing the survey, a pilot test was conducted with a small group of participants using the think-aloud protocol to ensure clarity of the survey and eliminate any ambiguity or unclear words and expressions. After gaining ethics approval from the Institutional Review Board (IRB) of the first author’s institution participants provided informed consent with the option to withdraw from the survey at any time. All the procedures were carried out following the Declaration of Helsinki. Attention checks were included in the survey to ensure data quality. Eligible participants received compensation for their participation and the study was conducted from mid-July 2022 to mid-February 2023.

To determine the appropriate sample size, we used Green’s formula.^57^ It suggests that a minimum sample size of 50 + 8 times the number of independent variables *p* is needed for a linear regression analysis. This indicates that a minimum sample size of 82 participants in each culture is adequate to examine the impact of our four independent variables. Moreover, according to MacKinnon et al,^58^ when testing various mediation analysis methods, they observed that the Type I error rates remained within the robustness interval for sample sizes of at least 100. Consequently, we aimed for a sample size of 250 participants in each group, supported by previous findings demonstrating correlation stability.^59^ The European dataset consisted of 262 participants (57.63% male, aged 18–66) and included individuals predominately from Germany (n = 122 Participants), and other European countries as specified earlier, representing a diverse range of professions, with 64.89% being employed (including self-employed), 29.00% being students, and 6.11% being unemployed. As for the Arab dataset, 251 participants were recruited (60.56% male, aged 18–59). Despite opening the survey to all Arab countries, we received responses from Egypt, Bahrain, Algeria, Iraq, Jordan, Kuwait, Lebanon, Morocco, Oman, Palestine, Saudi Arabia, Somalia, Sudan, Syria, Tunisia, UAE, and Yemen. The participants in this dataset also represented a diverse range of professions, with 67.73% employed, 22.71% students, and 9.56% unemployed participants.

### Measures

#### Demographic Measures

The participants provided information on age, gender, profession, nationality and country of residence.

#### Problematic Social Media Use (PSMU)

Problematic social media use was measured by employing both the original English version and a translated Arabic version of Social Media Disorder scale.^4^ The Arabic version of the scale was developed for the present study using the recommended back-translation method^60^ and administered by three of the authors who are bilingual and proficient in both languages. The scale includes nine items aligned with the diagnostic criteria for Internet Gaming Disorder as outlined in DSM-5. Each item corresponds to a specific diagnostic criterion of SMD, encompassing Preoccupation, Tolerance, Withdrawal, Persistence, Escape, Problems, Deception, Displacement, and Conflict. Participants rated each item on a 5-point Likert scale, ranging from “1 = Never” to 5 = Always”. The total score was obtained by summing the responses, with higher scores indicating a higher level of SMD. The scale has demonstrated good internal consistency with Cronbach’s alpha ranging from α = 0.76 to α = 0.82.^4^ In the present study, Cronbach’s alpha was α = 0.90 for the European sample and α = 0.85 for the Arab sample.

#### Need for Affect (NFA)

To assess individual differences in participants’ tendency to approach or avoid emotion-inducing situations, the present study employed the short version of the Need for Affect scale.^19^ For the European sample, the original English version was utilized, while for the Arab sample, a translated version in Arabic was used, following the aforementioned back-translation method. The 10-item short version of the scale was derived from the Need for Affect Questionnaire (NAQ) developed by Maio & Esses.^20^ This version encompasses two core subscales: affect approach and affect avoidance, each consisting of five items. Participants rated their agreement with each item on a 7-point Likert scale, ranging from “-3 = strongly disagree” to “3 = strongly agree”. The total score for each subscale was obtained by summing the responses for the corresponding items, with higher scores indicating higher tendencies in relation to approach or avoid affect. An example item for the approach facet of NFA is “I feel that I need to experience strong emotions regularly” and for the avoidance facet of NFA “I would prefer not to experience either the lows or highs of emotion”.^19^ The scale has demonstrated good internal consistency in the present sample with Cronbach’s alpha values of α = 0.78 for affect approach and α = 0.87 for affect avoidance in the European sample and α = 0.73 for affect approach and α = 0.82 for affect avoidance in the Arab sample.

#### Fear of Missing Out (FoMO)

To assess the concept of FoMO, a single-item scale was employed which was originally developed and validated by Riordan et al.^61^ It was utilized in its original English version for the European sample and a translated Arabic version was used for the Arab sample, following the established back-translation method. Participants were requested to assess their experience of FoMO by responding to the following item: “Do you experience FoMO (the fear of missing out)? FoMO refers to the fear of not being able to know what is happening online and participate in it”. Responses were recorded using a 5-point Likert scale, ranging from “1 = Not at all true of me” to “5 = Extremely true of me” with a higher score reflecting a more pronounced fear of missing out.

### Data Analysis

To prepare the data for analysis, 139 participants from the European sample and 476 from the Arab sample were excluded from the dataset due to their failure to pass multiple attention checks, providing contradictory responses, or leaving the survey incomplete (original sample size in Europe was 401 and original sample size in the Arab sample was 727 participants). Prior to conducting the analysis, we assessed all variables to ensure they met the normality assumptions. Skewness and kurtosis values as well as Q-Q plots were examined, and all variables demonstrated values within the acceptable range of less than ±1, indicating a normal distribution. Therefore, a parametric approach was adopted for further analysis. Additionally, outliers were identified by inspecting the boxplots of the variables, defined as scores that fell outside the boxplot whiskers determined by Tukey’s formula.^62^ Within the Arab sample, five outliers were detected for the variable affect approach while one outlier was detected in the European sample. These outliers (n = 6) were addressed by replacing them with the highest non-outlier scores observed in their respective samples. The results, with and without outlier replacement, exhibited slight and statistically insignificant changes, indicating minimal impact on the data. To identify gender differences within the European and Arab samples, as well as the overall differences between the two samples, independent sample *t*-tests were conducted (employing Welch’s *t*-test when necessary). Additionally, correlation analysis was performed to examine the associations among all variables: PSMU, FoMO, affect approach, affect avoidance, age and gender. Point-biserial correlation was utilized for gender, while Pearson’s correlation was employed for the remaining variables. Multiple linear regression analysis was employed to “predict” PSMU (operationalized with the SMD scale) from the variables affect approach, affect avoidance, age and gender with PSMU while ensuring that the assumptions of linearity, homoscedasticity, and multicollinearity were met. Subsequently, mediation analysis was conducted to examine the mediating effect of FoMO on the relationship between affect approach, affect avoidance and PSMU. The mediation analysis was conducted using standard method available in JASP software which employs the product of coefficient approach described in reference.^63^ This method is suggested to provide more accurate Type I error rates and robust statistical power. All analysis were performed using JASP software.^64^

## Results

### Descriptive Statistics

Table 1 presents the demographic characteristics of the participants, with a total of 513 participants, including 262 from European countries and 251 from Arab countries. Table 2 presents the descriptive statistics of PSMU, FoMO, affect approach and affect avoidance. Interestingly, the results showed significant gender-related association with PSMU, FoMO, and affect approach within the European sample with small to medium effect size, but not within the Arab sample.

**Table 1:** Participants Demographics

Variables	European N (262)	Arab N (251)
**Gender**		
Male (%)	151 (57.63)	152 (60.56)
Female (%)	111 (42.37)	99 (39.44)
**Age**		
M (*SD*)	29.16 (8.42)	31.24 (8.23)
Range	18–66	18–59
**Employment** (%)		
Student	76 (29.00)	57 (22.71)
Employed/Self-employed	170 (64.89)	170 (67.73)
Not employed	16 (6.11)	24 (9.56)

**Table 2:** Descriptive Statistics of PSMU, FoMO, Affect Approach and Affect Avoidance

	European Sample				Arab Sample				Differences Between the Two Samples
	Total Sample (N = 262)	Male (151)	Female (111)	Gender Differences	Total Sample (N = 251)	Male (152)	Female (99)	Gender Differences	Total Sample (N = 513)
PSMU	21.39 (7.44)	20.54 (7.95)	22.54 (6.55)	t(256.61)= −2.23, p=0.027, d=−0.27*	21.11 (6.51)	20.72 (6.59)	21.71 (6.38)	t(249.00)= −1.18, p=0.240, d= −0.15	t(506.87)= 0.46, p=0.648, d= 0.04*
FoMO	2.93 (1.19)	2.78 (1.22)	3.14 (1.12)	t(260.00)= −2.40, p=0.017, d=−0.30	2.24 (1.28)	2.30 (1.32)	2.15 (1.20)	t(249.00)= 0.92, p=0.360, d= 0.12	t(511.00)= 6.32, p<0.001, d= 0.56
Affect approach	0.88 (1.02)	0.66 (1.05)	1.18 (0.90)	t(253.67)= −4.29, p< 0.001, d=−0.53*	0.94 (0.94)	0.87 (0.99)	1.04 (0.85)	t(249.00)= −1.48, p=0.139, d= −0.19	t(511.00)= −0.64, p=0.520, d= −0.06
Affect avoidance	−0.55 (1.39)	−0.63 (1.34)	−0.44 (1.46)	t(260.00)=−1.05, p=0.296, d= −0.13	−0.19 (1.20)	−0.16 (1.14)	−0.23 (1.30)	t(249.00)= 0.43, p=0.671, d= 0.06	t(505.98)= −3.13, p=0.002, d= −0.28*

**Notes**: *Welch’s *t*-test.

### Correlational Analysis

The Pearson’s correlation analysis presented in Table 3 revealed significant associations between variables in the European and Arab samples. Specifically, in the European sample, significant associations were found between PSMU and both FoMO and affect avoidance. In the Arab sample, significant associations were found between PSMU, affect approach, affect avoidance, and FoMO.

**Table 3:** Pearson’s Correlation Analysis of Affect Approach, Affect Avoidance, FoMO, PSMU Age and Gender

Variable	PSMU	FoMO	Affect Approach	Affect Avoidance	Age	Gender
**PSMU**	–	0.45 ***	0.22***	0.29***	−0.11	0.07
**FoMO**	0.58***	–	0.27 ***	0.24 ***	−0.09	−0.06
**Affect approach**	0.11	0.16**	–	−0.03	−0.10	0.09
**Affect avoidance**	0.22***	0.18**	−0.33***	–	−0.06	−0.03
**Age**	−0.17**	−0.20***	−0.08	−0.24***	–	–
**Gender (Male)**^a^	0.13*	0.15*	0.26***	0.07	–	–

**Notes**: ^a^Point-biserial correlation. *p < 0.05, **p < 0.01, ***p < 0.001. European sample is presented below the diagonal, Arab sample is presented above the diagonal.

### Multiple Regression Analysis

Multiple regression analysis demonstrated that affect approach and affect avoidance predicted PSMU in both samples (European: *F* (4, 257) = 6.78, *p* < 0.001, *R^2^_adjusted_* = 0.08; Arab: *F* (4, 246) *=* 10.47, *p <* 0.001, *R^2^_adjusted_ =* 0.13). Within the model, for both European and Arab samples, affect avoidance demonstrated the strongest prediction for PSMU as indicated in Table 4.

**Table 4:** Multiple Regression Analysis for NFA Components, Age and Gender as Predictors of PSMU

Predictors	Europeans			Arabs		
	β	*t*	*p*	β	*t*	*p*
Affect approach	0.17	2.52	0.012	0.22	3.61	< 0.001
Affect avoidance	0.25	3.79	< 0.001	0.29	4.98	< 0.001
Age	−0.09	−1.40	0.164	−0.07	−1.24	0.215
^a^Gender	0.06	0.93	0.354	0.06	1.08	0.281

**Note**: ^a^Male: 0, Female: 1.

### Mediating Effect of FoMO

**Figure 1:**
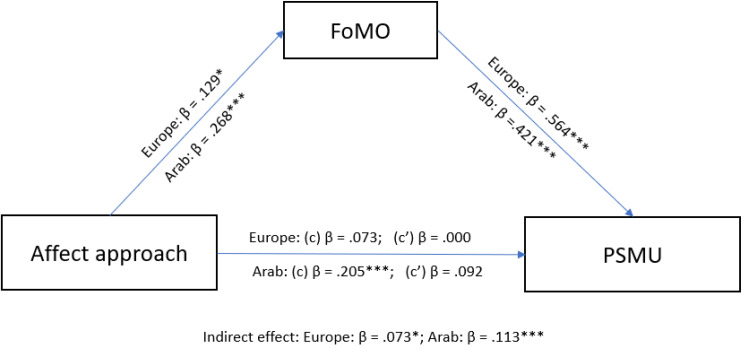
Mediation model between affect approach and problematic social media use through FOMO: (c) Total effect, (c’) Direct effect. *p < 0.05; **p < 0.01; ***p < 0.001.

**Figure 2:**
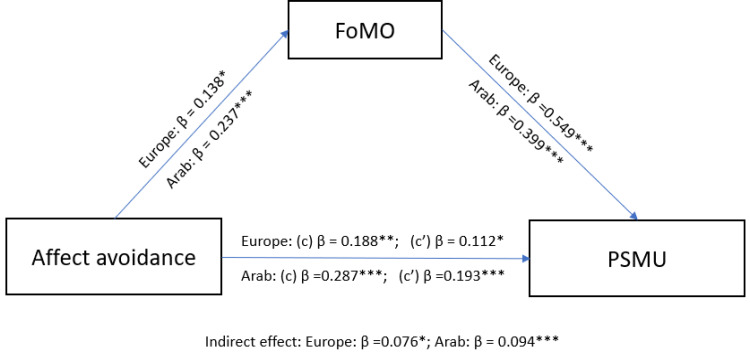
Mediation model between affect avoidance and problematic social media through FOMO: (c) Total effect, (c’) Direct effect. *p < 0.05; **p < 0.01; ***p < 0.001.

Following the significant results from correlation and regression analysis, mediation analysis was conducted to examine the mediating effect of FoMO in the relationship between affect approach (from the NFA scale), affect avoidance (from the NFA scale) and PSMU (hence the SMD scale). To account for the potential effect of age and gender, these variables were included as covariates in the analysis. For the European sample, the mediation model results showed non-significant total effect of affect approach on PSMU (*β* = 0.073, *SE* = 0.062, *p* = 0.242) but significant total effect of affect avoidance (*β* = 0.188, *SE* = 0.061, *p* = 0.002). The direct effect was not significant for affect approach (*β* = 0.000, *SE* = 0.052, *p* = 0.995) but was significant for affect avoidance (*β* = 0.112, *SE* = 0.052, *p* = 0.031). The indirect effect of FoMO was significant for affect approach (*β* = 0.073, *SE* = 0.035, *p* =0.040) and affect avoidance (*β* = 0.076, *SE* = 0.034, *p* = 0.028). These findings indicate that FoMO partially mediated the relationship between affect avoidance and PSMU in the European sample (Figures 1 and 2). In the Arab sample, the mediation model results showed a significant total effect of affect approach on PSMU (*β* = 0.205, *SE* = 0.062, *p* < 0.001) and for affect avoidance on PSMU (*β* = 0.287, *SE* = 0.060, *p* <0.001), a non-significant direct effect for affect approach (*β* = 0.092, *SE* = 0.058, *p* = 0.116) but significant direct effect for affect avoidance (*β* = 0.193, *SE* = 0.056, *p* <0.001), and a significant indirect effect via FoMO for affect approach (*β* = 0.113, *SE*= 0.030, *p* < 0.001) and affect avoidance (*β* = 0.094, *SE* = 0.028, *p* < 0.001). These findings indicate that FoMO fully mediated the relationship between affect approach and PSMU, while also playing a partial mediating role in the relationship between affect avoidance and PSMU (Figures 1 and 2).

## Discussion

Existing research findings highlight the widespread and integral role of social media in people’s lives, while also acknowledging particular concerns about problematic social media use (here assessed with the SMD scale) and its potential detrimental consequences. Social media serves as a unique platform for meeting opposing needs, such as approaching positive emotions or avoiding negative ones. Positive emotions, eg, excitement, are often harnessed to enhance user engagement and encourage interaction.^65^,^66^ On the other hand, social media can also serve as a medium for escapism, allowing users to seek relief from negative emotions, loneliness^67^ and enhance mood.^68^

To deepen our understanding of the underlying affect factors contributing to PSMU, this study examined the role of the NFA, namely affect approach and affect avoidance, on PSMU. We also tested the mediating effect of FoMO in this relationship. The findings confirmed both of the proposed hypotheses, consistent with the research on concepts usually associated with NFA, such as neuroticism in the context of FoMO and Internet Addiction,^69^ and possibly others, such as sensation seeking in other maladaptive behaviors.

A significant difference was observed in the descriptive statistics of the variables under study. The European sample exhibited a higher level of FoMO compared to the Arab sample, possibly reflecting potential cultural differences. The result is somewhat surprising, as scores in Uncertainty Avoidance, a cultural dimension closely related to FoMO, as indicated by Hofstede Insights’ index,^70^ are somewhat similar between Arabs and Europeans (the majority of our sample, as indicated in the dataset section, comprises Germans in particular). However, Arabs scored significantly higher in Collectivism as a cultural dimension. In societies with a strong emphasis on Collectivism, like Arab countries, there is a tendency to prioritize social bonds and embrace interdependence and conformity.^71^ As a result, these societies may experience lower levels of FoMO, owing to the preexisting measures in place to maintain relationships. Another possible reason could also be related to the amount of content exposed by Arabs online. Privacy has been shown to be higher in conservative societies such as Arabs,^72^ which can lead to less content exposure and, consequently, lower FoMO. Interestingly, European females demonstrated higher scores of affect approach, FoMO and PSMU than European males. This is consistent with previous findings^46^,^49^,^51^ but contradictory to studies who found no gender-specific differences on Social Media Use (SMU)^73^ and FoMO^33^ or higher scores of SMU and FoMO across males.^45^,^74^ This also suggests that the literature is inconsistent, and there is a need to unified studies design and to consider an additional set of variables that may influence the relationship between gender and FoMO and PSMU. For example, the type of online forums used and the use of a real versus a made-up identity may have an impact on the feeling of FoMO.

The significant correlation results within the Arab sample suggest that higher levels of PSMU were associated with increased FoMO and stronger tendencies towards both affect approach and affect avoidance. Moreover, a significant positive correlation was observed between FoMO, affect approach and affect avoidance in both samples, indicating that individuals with higher FoMO also tended to have stronger tendencies towards affect approach and affect avoidance. Affect approach and affect avoidance exhibited a significant negative association in the European sample, while no significant association was observed in the Arab sample. Additionally, PSMU displayed a significant association with affect avoidance in the European sample, and a significant association with both affect avoidance and affect approach in the Arab sample, suggesting potential cultural differences in emotional patterns highlighting how these cultural disparities in emotions emerge as a function of people’s ongoing social interactions and relationships.^75^ In individualistic cultures, where high arousal emotions such as excitement, fear and joyful are valued,^76^ individuals might be more inclined to experience and exhibit behaviors associated with FoMO. The pursuit of excitement and novelty could lead individuals from these cultures to experience a heightened FoMO compared to societies higher in collectivism where a greater value is placed on low arousal emotions contributing to less pronounced experience of FoMO.^75^ Its noteworthy that there may be other factors contributing to the variations in results. For example, while the age and gender distributions were similar in both samples, the European sample had a slightly higher percentage of students compared to the Arab sample.

The regression analysis revealed that both elements of NFA predicted PSMU, although the affect avoidance was a stronger predictor of PSMU for both European and Arab samples. This observation aligns with Marino et al’s^30^ proposition wherein the motive for amplified positive affect and motive for reduced negative affect can explain PSMU. Our findings are also consistent with Andreassen’s findings^77^ showing that compulsive social media usage is driven by positive outcomes such as receiving feedback from others (see also a recent study on Likes by Marengo et al^78^ as well as avoiding negative consequences such as boredom (see also a recent work on boredom proneness)).^79^ This finding posits the implication that social media users should be encouraged to reflect on their motives behind their own social media use and their attitudes towards emotions. Particularly, the pursuit of positive emotional experiences or avoidance of negative ones through social media use may potentially lead to problematic social media use, here operationalized as addictive behavior with the SMD. Although the European sample displayed gender differences in PSMU, the Arab sample showed contrasting results. However, the regression analysis yielded insignificant results for both age and gender in both samples, indicating that they are not significant predictors of PSMU. Therefore, it can be suggested that gender and age do not have a substantial impact on the development of PSMU which is consistent with the study conducted by Aydin et al,^80^ that found no significant gender effect in “social media addiction” among a sample of 419 participants. Additionally, Huang^81^ reported a non-significant effect of age on problematic social media usage.

In the European sample, the mediation analysis showed a significant indirect association between affect approach and PSMU, through FoMO, while also indicating a partially mediating effect of FoMO in the relationship between affect avoidance and PSMU. In the Arab sample, FoMO partially mediated the effect of both affect approach and affect avoidance on PSMU. These results suggest that FoMO plays a significant mediating role in the relationship between the components of need for affect and PSMU, with varying degrees of mediation observed across the two cultural samples. Both escaping negative emotions and seeking positive emotions are associated with excessive social media use according to Andreassen’s findings^77^ and the partial mediation of FoMO on the relationship between affect avoidance and PSMU in the present samples suggest that not only is FoMO a potential factor associated with PSMU, but also the avoidance or escape from emotion-inducing situations contributes to PSMU.

The results suggest that the inclination towards affect avoidance is more pronounced, aligning with previous research on “SNS addiction”,^77^ which proposed that individuals might develop habitual or excessive social media use as a means to escape negative moods. Moreover, other studies have found that emotional stability indirectly influences problematic Facebook use through coping and conformity mechanisms.^30^ Social media platforms may serve as outlets for emotional frustration, leading to an association between heightened problematic social media use and emotion dysregulation through FoMO.^14^ Emotional reactions can happen more quickly and confidently compared to cognitive judgments,^82^ suggesting that affective associations with social media usage might be more immediate and automatic. Positive or negative emotional reactions can occur without extensive cognitive processing; therefore, they may influence individuals’ social media behaviors in a rapid and intuitive manner, potentially leading to impulsive and habitual usage patterns. This is particularly relevant when individuals find relief through social media. A longitudinal study showed that initial levels of social media use to alleviate boredom were linked to problematic social networking site use, financial stress, anxiety, and empathy.^83^ This usage also showed a significant positive association with negative emotions among medical college students during the COVID-19 pandemic^84^ with FoMO mediating the relationship between social media usage and negative emotions. However, research suggests that individuals might overestimate the positive emotional effect they expect to experience from social media usage.^85^ The quick and confident nature of emotional reactions could amplify this effect, as individuals might impulsively turn to social media seeking relief or positive emotions, only to end up feeling worse afterward. This discrepancy between expectations and actual emotional experiences may contribute to the development of social media disorder or excessive use, as individuals continue to engage with the platform in search of the positive emotional outcomes they anticipate, despite the negative consequences they encounter.

While social media offers a valuable means for social interaction and stress relief, it can also foster problematic behaviors, particularly among individuals with psychological needs like the pursuit of positive experiences or avoidance of negative ones. This parallels, for instance, the current educational emphasis on creating enjoyable and fun classrooms, which, if not properly managed, could impede effective learning. In line with this, a longitudinal study revealed that students in the gamified course exhibited declining motivation, satisfaction, and empowerment over time, unlike their peers in non-gamified class.^86^ This might be a result of the Goodhart’s Law which states that when a measure becomes a target, it ceases to be a good measure.^87^ It could be also related to the known fact that intrinsic motivation decreases when the behavior is measured and extrinsically motivated.^88^ Therefore, to address the complexities of social media’s emotional impact, we highlight the importance of digital wellbeing services which can empower users to set emotional and intentional goals. This could be accomplished by setting goals and limits not only in terms of usage amounts but also by considering mode, intention, and emotional states to manage both approach and avoidance.

Our study opens avenues for further investigation of the impact of the need for affect on PSMU and the development of effective digital wellbeing strategies. The fact that both affect approach and affect avoidance predict PSMU suggests that social media can fulfill both of these needs on a single platform simultaneously. This complexity makes it challenging to discern when and why people use it for one need over the other. This study contributes to the existing literature by examining generalizability of the mediating effect of FoMO on the relationship between affect approach, affect avoidance and PSMU across diverse cultural contexts.

## Limitations

This study has some limitations that may impinge the interpretations of our findings and the quality and replicability of the research. The study design is cross-sectional, and thus we cannot establish causality based on our findings. This can be more noticeable in mediation analysis, which implies the influence of the predictor on the mediator, which then affects the outcome. Another limitation arises from the use of self-report measures and the possibility of common sources bias. This risk was mitigated through the anonymous data collection procedures and the freedom given to participants to withdraw at any time. We have also assessed the internal reliability of the already validated scales we used, and our data showed a very good level of reliability. That said, it is worth noting the inherent limitations of self-reported data in terms of accuracy and subjectivity. Additionally, while our sample size was in line with recommendation from prior research regarding statistical analysis needs, in future studies, an increased sample size could enhance result replicability and ensure a nationally representative sample, as long as the survey dissemination is conducted meticulously to reach various sectors and geographic regions. Therefore, it’s essential to consider our study as exploratory, rather than representative of the entire populations. The fact that the results were largely similar between the Arab and European populations provides evidence of data quality and suggests a higher likelihood that the results would remain consistent with a larger sample size.

This study is innovative in exploring emotional states and how through their effect on FoMO can potentially lead to PSMU (but note that also FoMO as a trait closely linked to neuroticism,^33^ could be a vulnerability factor). It is imperative that future research explores whether the way that individuals engage with social media (passive versus active social media use) impact the association between NFA and PSMU. Future studies may also explore the potential of personalized content curation in social media to optimize positive emotions while minimizing potential negative effects.

## Conclusion

In conclusion, this study delved into the intricate relationship between emotional factors, problematic social media use, and its potential implications, shedding light on the nuanced dynamics of individuals’ interactions with digital platforms. The findings underscore the multifaceted role of social media as both a source of positive emotional experiences and an avenue for escaping negative emotions. Our results in two cross-cultural samples suggest that both affect approach and affect avoidance contribute significantly to the development of PSMU. Additionally, FoMO served as a partial mediator between affect approach and PSMU solely in the Arab sample, while it partially mediated the relationship between affect avoidance and PSMU in both cultural groups. This study highlights the need for digital wellbeing strategies that empower users to navigate their emotional inclinations in a manner that foster positive experiences while mitigating the risks of excessive usage. Moreover, it emphasizes the promising prospect of managing FoMO to enhance emotional control, thereby limiting the development of problematic social media use.

## Data Sharing Statement

The dataset associated with this work are uploaded alongside this article as Supplementary Material.
